# 中国多发性骨髓瘤自体造血干细胞移植指南（2021年版）

**DOI:** 10.3760/cma.j.issn.0253-2727.2021.05.001

**Published:** 2021-05

**Authors:** 中华医学会血液学分会浆细胞疾病学组 中国医师协会多发性骨髓瘤专业委员会

多发性骨髓瘤（multiple myeloma，MM）的移植至今经历了自体造血干细胞移植（autologous stem cell transplantation，auto-HSCT）、异基因造血干细胞移植（allogeneic hematopoietic stem cell transplantation，allo-HSCT）、auto-HSCT序贯allo-HSCT等。由于MM患者中位诊断年龄较大等原因，allo-HSCT未能作为大部分新诊断MM患者的首选[1]。auto-HSCT序贯allo-HSCT是先行auto-HSCT以减少肿瘤负荷，续贯allo-HSCT（通常减低剂量），对其疗效的报道相互矛盾[2]，所以临床未能广泛应用。因此，至今为止，auto-HSCT仍是适合移植MM患者的主要治疗方式。

自20世纪80年代初auto-HSCT开始应用于MM的治疗以来，患者的总生存（OS）期明显延长，因此，auto-HSCT一直被认为是年龄≤65岁新诊断MM患者的首选治疗选择[3]。但随着新药的不断涌现，MM的疗效得到很大提高，含新药方案4～6个疗程的完全缓解（CR）率即可达20％～40％，因此，auto-HSCT在MM治疗中的地位曾被质疑。但一代新药及二代新药诱导治疗后序贯auto-HSCT及持续新药治疗的前瞻（平行）对照研究的临床结果表明，经auto-HSCT治疗的MM患者无进展生存（PFS）获益更多，说明auto-HSCT在新诊断MM患者中治疗地位的重要性[4]。即使应用含单克隆抗体的方案作为诱导治疗，auto-HSCT仍是适合新诊断MM患者的重要治疗手段[5]。

我国MM患者平均诊断年龄较欧美国家年轻10岁左右，但接受auto-HSCT的比例却明显低于欧美国家[6]，这种现象可能是导致我国MM患者PFS和OS明显劣于欧美国家的原因之一。因此，有必要制定针对我国MM患者的auto-HSCT指南，以规范和指导我国MM患者的auto-HSCT治疗。

一、移植患者的筛选和要求

1. 年龄：一般而言，auto-HSCT在65岁以下且无严重脏器功能障碍的患者中进行，但auto-HSCT的年龄上限在国际上逐渐放宽。大于65岁的体能状态佳（fit）的MM患者实施auto-HSCT也可使PFS和OS获益，且未显著增加移植相关死亡率[7]。但对于有合并症的MM患者，接受auto-HSCT有增加移植不良反应和相关死亡率的风险，因此65岁以上MM患者实施auto-HSCT应在经验丰富的治疗团队进行仔细的体能状态评估后，在评分为fit的患者中进行。

2. 肾功能：肾功能损害是MM患者常见的临床表现，诱导治疗后部分患者肾功能可完全恢复正常或明显改善，不影响移植。即使不能完全恢复甚至仍需血液透析，也并非接受auto-HSCT的禁忌证。但肾功能不全会使移植相关不良反应如黏膜炎、感染等并发症增加，因此，需要降低预处理药物的剂量[8]。

3. 其他：肺部感染也是MM常见的临床表现，诱导治疗有效的MM患者肺部感染会明显减轻或完全好转，不影响移植的进行，但若患者有慢性阻塞性肺疾病或其他导致肺功能严重下降的疾病时，auto-HSCT前需评估肺功能。当肺功能中一秒用力呼气容积（forced expiratory volume in one second，FEV1）占预计值百分比<60％和（或）弥散功能占预计值百分比<60％时，暂不宜行auto-HSCT[9]。患者原有心脏疾病或因继发淀粉样变性导致心功能不全时，需充分改善心功能，达到一定条件才能行移植，这些条件包括肌钙蛋白T（TnT）<0.06µg/L、收缩压≥90 mmHg（1 mmHg＝0.133 kPa）、美国纽约心脏病协会（NYHA）分级1～2级[10]。

二、移植前的诱导治疗

移植前MM患者需行诱导治疗以尽快减轻肿瘤负荷，恢复脏器功能。新诊断MM患者诱导治疗的药物包含以下几类：蛋白酶体抑制剂（硼替佐米、伊沙佐米等）；免疫调节剂（沙利度胺、来那度胺等）；单克隆抗体（抗CD38单克隆抗体等）；细胞毒药物（环磷酰胺、脂质体阿霉素等）；糖皮质激素（地塞米松、泼尼松等）等。新诊断适合移植患者的诱导方案目前以三药联合为主，包括：硼替佐米+来那度胺+地塞米松（VRD）、硼替佐米+沙利度胺+地塞米松（VTD）、硼替佐米+环磷酰胺+地塞米松（VCD）、伊沙佐米+来那度胺+地塞米松（IRD）、硼替佐米+脂质体阿霉素+地塞米松（PAD）、沙利度胺+阿霉素+地塞米松（TAD）、沙利度胺+环磷酰胺+地塞米松（TCD）等[11]。国际上已有推荐在三药基础上联合单克隆抗体（如抗CD38单克隆抗体）的诱导治疗方案，目的是提高移植前的疗效[5]。

拟行auto-HSCT的MM患者诱导药物的选择需注意避免对造血干细胞的毒性蓄积作用，避免影响造血干细胞的采集和造血重建。蛋白酶体抑制剂、沙利度胺、单克隆抗体及糖皮质激素均不损伤造血干细胞，但随着化疗疗程数的增加，来那度胺及烷化剂等细胞毒药物对正常造血干细胞的损伤可能也增加，因此一般建议应用含此类药物的化疗不超过4个疗程即进行造血干细胞采集[12]。诱导化疗前还应评估是否有心肌淀粉样变性及其严重程度，如有心肌淀粉样变性，应避免使用心肌毒性药物；伴肾功能不全者建议使用含硼替佐米的联合方案，如使用来那度胺应根据肌酐清除率调整药物剂量；伴髓外浆细胞瘤的MM患者建议使用含细胞毒药物的多药联合化疗。造血干细胞动员前诱导化疗的疗程数大多为4个疗程，超过6个疗程并不明显增加缓解深度。

三、移植时机的选择

早期移植指诱导治疗缓解后立即移植，即移植在诊断后1年内进行。晚期移植是诱导治疗后先采集造血干细胞冻存备用，随后予药物巩固维持治疗，至首次复发后再进行移植。虽然OS时间可能相似，但早期行auto-HSCT患者的PFS时间较晚期行auto-HSCT患者更长，患者的生活质量更高[13]。因此认为早期auto-HSCT是符合移植条件的MM患者的标准治疗，特别是诱导治疗后微小残留病（MRD）未转阴的高危和标危MM患者。目前研究证明，对于诱导治疗后MRD转阴的标危MM患者，晚期auto-HSCT也是可行的，但应告知计划行晚期auto-HSCT的患者，可能有25％的患者在未来疾病复发时因各种原因无法实施auto-HSCT。

一般而言，诱导治疗的缓解深度对auto-HSCT后患者的预后有影响。诱导治疗缓解程度越深，移植后的PFS时间和OS时间越长，尤其是获得MRD阴性的患者[14]。但对于部分仅获得疾病稳定（SD）或微小缓解（MR）的MM患者，由于后续造血干细胞采集及预处理采用大剂量细胞毒药物，有别于诱导治疗应用的蛋白酶体抑制剂和（或）免疫调节剂，因此即使诱导治疗4个疗程后未能获得非常好的部分缓解（VGPR）及以上疗效，也可能从auto-HSCT中获益。诱导治疗后未获得VGPR及以上疗效的患者也可选择其他治疗方法。

四、自体造血干细胞的动员、采集和保存

外周血造血干细胞动员方法包括粒细胞集落刺激因子（G-CSF）单药或联合普乐沙福及大剂量化疗联合G-CSF。G-CSF以5～10 µg·kg^−1^·d^−1^应用5～7 d，普乐沙福是趋化因子受体CXCR4的抑制剂，能够增强G-CSF的干细胞动员作用。单用G-CSF或G-CSF联合普乐沙福动员方案的优点是采集时间易预测且耐受性良好，可门诊用药，但对于疗效缓解欠佳的MM患者，该方法不能进一步减少肿瘤负荷，还可能加重原发病；同时，单用G-CSF的患者采集的细胞数有限，采集的失败率较高。大剂量化疗联合G-CSF的动员方案常用3～5 g/m^2^的环磷酰胺，也有研究采用依托泊苷、阿糖胞苷、苯达莫司汀和DECP（地塞米松+依托泊苷+环磷酰胺+顺铂）方案作为动员方案。大剂量化疗联合G-CSF的动员方案采集时间通常在动员开始后第10～13天，该方案的优点是采集的成功率高，造血干细胞数量多，且可进一步提高诱导治疗后疗效不理想患者的疗效。但也存在需要住院、化疗相关不良反应、治疗风险及费用增加等缺点。

以化疗为基础的动员在WBC降至最低又重新上升至（2～4）×10^9^/L或单核细胞比例20％～40％时行外周血造血干细胞采集，也可通过检测外周血CD34阳性细胞数作为开始采集及预测采集是否成功的标记。以化疗为基础的动员在第8～10天、G-CSF单药或联合普乐沙福动员的第4或5天开始进行外周循环血中CD34细胞的监测，达到10个/µl可作为采集的阈值，达到20个/µl容易获得采集成功[15]。

干细胞采集前需评估患者外周静脉条件，必要时开放中心静脉通路，循环血量一般按患者血容量的2～3倍计算（患者血容量按70 ml/kg），一次动员采集天数（次数）一般不超过4 d（或4次）。干细胞保存需要在有资质的单位进行，一般采用细胞冷冻保护剂二甲基亚砜（DMSO）进行干细胞的低温保存，采集后的干细胞加入含10％DMSO的细胞营养液中，分装于血液冻存袋内，经程控冷冻系统降温至−80°C，再投入液氮（−196°C）贮存，条件好的单位可保存8年或以上，也可采用−80°C低温冰箱冷冻法。单次auto-HSCT需要采集的CD34^+^细胞数最好大于2×10^6^/kg。建议在第一次动员时即采集满足两次auto-HSCT所需的造血干细胞数量，为高危患者的双次移植或标危患者进行挽救性二次移植储备两次移植所需的干细胞。

造血干细胞采集失败的原因与患者年龄、诱导治疗选用的药物和（或）疗程数等有关。首次动员采用单药G-CSF方案失败者，为增加动员的成功率，如全身情况允许，可改用大剂量化疗联合G-CSF动员方案或G-CSF联合普乐沙福方案作为补救。如采集的干细胞数过少，可采用外周血联合自体骨髓移植或自体骨髓移植。

对于动员后直接进入层流仓进行预处理的患者，如用美法仑预处理方案，48 h内（需在预处理结束后24 h）即可回输干细胞，由于储存时间短，干细胞可保存在4°C冰箱中（要求在48 h内回输），回输前干细胞不需作特殊处理。对于虽使用美法仑作预处理但采集后不立即进仓进行预处理的患者（大部分患者采用此方式），或预处理方案采用美法仑以外需连用7 d或以上药物的患者，其干细胞需储存于−80°C冰箱或液氮。

五、预处理和造血干细胞回输

美法仑是MM患者auto-HSCT预处理方案中使用最多的药物，美法仑200 mg/m^2^被推荐为MM患者的标准预处理方案[16]。为减少移植相关并发症和死亡率，对于肾功能不全（血清肌酐清除率<60 ml/min）的患者，美法仑剂量应减至140 mg/m^2^［8]。除了大剂量美法仑，CVB方案（环磷酰胺50 mg/kg，每日1次，−3～−2 d；依托泊苷10 mg/kg，每日1次，−5～−4 d；白消安0.8 mg/kg，每6 h 1次，−8～−6 d）[17]、BUCY方案（白消安0.8 mg/kg，每6 h 1次，−7～−4 d；环磷酰胺60 mg/kg，每日1次，−3～−2 d）等其他方案也在临床中选择使用。

预处理前需应用止吐药，并需充分碱化、水化、降尿酸，丙戊酸钠或苯巴比妥预防癫痫。造血干细胞输注前需要进行造血干细胞解冻、复苏。造血干细胞输注时需预防与DMSO输注相关的并发症，如应用苯海拉明、糖皮质激素预防DMSO的过敏反应。

六、移植后造血和免疫重建的监测

1. 造血重建的监测：造血重建监测的主要指标包括外周血中性粒细胞绝对计数（ANC）和血小板计数（PLT）。停用G-CSF后，ANC>0.5×10^9^/L连续3 d即达到粒系重建的标准。当脱离输注血小板时，能保持PLT>20×10^9^/L连续7 d即达到巨核系重建标准。大部分患者在造血干细胞回输后2周左右造血重建，但对于第二次移植患者，其造血重建可能会延迟。超过28 d仍未达到以上标准之一，称为造血重建延迟，主要表现为血小板延迟恢复时，可应用促血小板生成药物治疗。造血重建延迟可影响维持治疗开始或导致维持治疗中断，疾病复发风险可能增加。患者出院后应每1～2周复查血常规，观察ANC及PLT变化。如患者移植3个月后不能完成造血重建，需警惕疾病复发可能。

2. 免疫重建的监测：auto-HSCT后的免疫重建包括细胞免疫及体液免疫的重建，有条件的医院可进行免疫重建的监测，可定期复查T细胞亚群和T细胞、B细胞功能。移植后6个月IgG先恢复，IgA和IgM的恢复可能需要1～2年或更久，如auto-HSCT后1年内免疫抑制能恢复，往往提示患者的预后更好[18]。需要强调的是，移植后患者发生体液免疫重建时，某些患者外周血中可能会出现一过性单克隆免疫球蛋白，应与疾病复发鉴别。免疫重建的单克隆免疫球蛋白可以与初诊时单克隆免疫球蛋白相同，也可能不同，数量很低，不会引起其他免疫球蛋白进行性下降，骨髓流式细胞学检测无克隆性浆细胞，κ、λ轻链往往同时升高（此变化与初诊时单个轻链升高有显著差别），随访3～6个月或更久后会消失，这类患者往往预后较好。

七、auto-HSCT后的巩固与维持治疗

1. 巩固治疗：auto-HSCT后使用与原有效诱导化疗方案相同或相似的方案继续治疗2～4个疗程称为巩固治疗。对于非高危且auto-HSCT后获得CR或以上疗效的患者，可不进行巩固治疗。

2. 维持治疗：auto-HSCT患者在移植后无论是否巩固治疗均应进入维持治疗。既往维持治疗常应用化疗、干扰素及糖皮质激素等，由于疗效不确切，目前不再推荐。目前常用于维持治疗的药物包括沙利度胺、来那度胺、伊沙佐米和硼替佐米[19]。其中沙利度胺不建议用于伴高危细胞遗传学异常的患者，对于细胞遗传学标危的患者，沙利度胺仍可作为维持治疗药物之一，推荐剂量每晚100～200 mg。细胞遗传学标危及中危患者应用来那度胺的维持治疗获益更多，推荐剂量是10 mg/d，肾功能损伤患者应用来那度胺需调整剂量。对于伴高危细胞遗传学患者，建议采用硼替佐米单药或联合用药，一般每2～3个月为1个疗程。伊沙佐米维持治疗的剂量是4 mg（有肾功能损害者减少至3 mg），每个月的第1、8、15天使用。维持治疗持续至少2年。

八、移植后疗效监测和管理

移植后的第一年每3个月进行一次疗效评估，第二年起每6个月一次。如患者疾病指标不稳定，需缩短两次评估的间隔时间。监测指标包括血常规、肝肾功能（包括白蛋白、球蛋白、肌酐、β_2_-微球蛋白、乳酸脱氢酶）、电解质（包括钙离子）、血清和（或）尿M蛋白（蛋白电泳、免疫固定电泳）、血清或尿免疫球蛋白定量（包括轻链）、血清游离轻链（尤其是寡分泌型MM患者）、24 h尿轻链和骨髓检查。其他检查如骨骼X线检查、全身低剂量CT、骨骼MRI和（或）全身PET/CT可根据病情需要进行。监测过程中如出现临床复发，需启动复发后治疗。对于仅有生化复发的患者，受累单克隆免疫球蛋白上升速度缓慢，可先观察，待指标符合复发治疗指征时启动治疗，也可提早进入临床试验。如果出现单克隆免疫球蛋白升高速度加快（如3个月内增加1倍）时，需尽快启动治疗。维持治疗过程中微小残留病由阴转阳是否有指导复发治疗的意义目前尚无定论。

九、双次auto-HSCT在高危MM患者中的应用

在第一次auto-HSCT后的6个月内进行计划中的第二次auto-HSCT为双次移植或串联移植（tandem transplantation）。新药时代，二次移植不再根据第一次移植后的疗效决定，而是在具有高危因素的MM患者中进行[20]。高危MM患者在第一次移植后无论获得何种疗效，均建议在半年内进行第二次移植。需强调，计划双次移植的患者首次诱导治疗4个疗程后即采集两次移植所需的干细胞，两次移植之间不进行巩固和维持治疗。第二次移植采用的预处理方案美法仑剂量为140～200 mg/m^2^。

十、挽救性二次auto-HSCT在MM患者中的应用

MM患者选择auto-HSCT后，即使后续进行了规范的巩固和维持治疗，但每年仍然以10％～15％的比例复发，复发后再进行auto-HSCT即为挽救性二次移植。如在首次诱导治疗后即采集两次移植所需的干细胞，挽救性二次移植是一种安全有效的治疗方法[21]。首次移植后PFS时间越长，二次移植后的疗效越好。目前认为，第一次移植后PFS时间在2年以上、有足够的干细胞、体能状态佳的MM患者可考虑挽救性二次移植。挽救性二次移植前需进行再诱导治疗，有效后再进行挽救性二次auto-HSCT。不建议第一次移植后复发再行造血干细胞动员，第一次移植应用大剂量环磷酰胺动员、美法仑预处理并进行来那度胺维持治疗，往往导致动员失败。预处理方案仍可选择大剂量美法仑（200 mg/m^2^）。需注意挽救性二次移植后的造血重建可能会延迟。
